# Homogenization and impoverishment of taxonomic and functional diversity of ants in *Eucalyptus* plantations

**DOI:** 10.1038/s41598-018-20823-1

**Published:** 2018-02-19

**Authors:** Felipe Martello, Francesco de Bello, Maria Santina de Castro Morini, Rogério R. Silva, Débora Rodriges de Souza-Campana, Milton Cezar Ribeiro, Carlos P. Carmona

**Affiliations:** 1 Departament of Environmental Science, Federal University of São Carlos, UFSCar, Rod., Washigton Luís Km 235, São Carlos, SP Brazil; 2 Department of Botany, Faculty of Science, University of South Bohemia, Branišovská 31, České Budějovice, Czech Republic; 3 Laboratório de Mirmecologia do Alto Tietê (LAMAT), Universidade de Mogi das Cruzes, UMC, Avenida Dr. Cândido Xavier de Almeida Souza, 200, Centro Cívico, Mogi das Cruzes, São Paulo Brazil; 4 Museu Paraense Emílio Goeldi, Coordenação de Ciências da Terra e Ecologia, Av. Perimetral 1901, CEP 66077-830 Belém, PA Brazil; 5 Spatial Ecology and Conservation lab (LEEC), São Paulo State University, UNESP, Ecology Department, Avenida 24 A, 1515, Bela Vista, Rio Claro, São Paulo Brazil; 6 Institute of Ecology and Earth Sciences, University of Tartu, Lai 40, Tartu, 51005 Estonia

## Abstract

Despite its negative impacts on the environment and biodiversity, tree plantations can contribute to biodiversity conservation in fragmented landscapes, as they harbor many native species. In this study, we investigated the impact of *Eucalyptus* plantations on the taxonomic and functional diversity of ant communities, comparing ant communities sampled in managed and unmanaged (abandoned for 28 years) *Eucalyptus* plantations, and native Atlantic rain forests. *Eucalyptus* plantations, both managed and unmanaged, reduced the functional diversity and increased the similarity between ant communities leading to functional homogenization. While communities in managed plantations had the lowest values of both taxonomic and functional ant diversities, ant communities from unmanaged plantations had similar values of species richness, functional redundancy and Rao’s Q compared to ant communities from forest patches (although functional richness was lower). In addition, communities in unmanaged *Eucalyptus* plantations were taxonomically and functionally more similar to communities located in managed plantations, indicating that *Eucalyptus* plantations have a severe long-term impact on ant communities. These results indicate that natural regeneration may mitigate the impact of *Eucalyptus* management, particularly regarding the functional structure of the community (α diversity), although it does not attenuate the effects of long term homogenization in community composition (β diversity).

## Introduction

The conversion of native habitats into human-modified land use reduces and fragments habitats into small remnants and is one of the main drivers of biodiversity loss^1^. Among these conversions, tree plantations (especially *Eucalyptus, Acacia* and *Pinus*) have become one of the fastest growing land uses in recent decades, mainly to attend the high demand for timber and cellulose^2^. Brazil has the largest area of *Eucalyptus* plantations in the world, totaling about 7.74 million hectares, and is the largest exporter of *Eucalyptus* cellulose^3^. These plantations –located mostly in areas originally covered by rain forest– are extensive monocultures that are managed through ploughing, fertilizers and herbicides, and are clear cut harvested after 5–10 years of planting^3^. *Eucalyptus* plantations, like other tree plantations, have been the focus of a vigorous debate about their impact on conservation. This debate is particularly intense in regard to plantations located in heterogeneous landscapes composed of native forest patches and anthropic land uses without a forest-vegetation phytophysiognomy^2^. *Eucalyptus* plantations may harbor native species more effectively than non-forest anthropic land uses^4^ and can also increase landscape connectivity, *i.e*., the dispersion and movement of native species^5^. Besides their commercial use, less intensively managed tree plantations may also be established to reduce erosion, combat global warming through carbon sequestration, and facilitate land and vegetation rehabilitation^2,6^. On the other hand, the high fertilizer and water demands, the release of allelopathic substances, and the intensive management – all of which are characteristic of industrial-scale *Eucalyptus* plantations– severely impact soil structure, water supply, and reduce the stability and biodiversity of tree plantations relative to native forests^6–8^. The effect of *Eucalyptus* plantations on biodiversity also reflects this ambiguous nature of tree monocultures: diversity (richness and abundance) can change or remain largely unchanged for many organisms. For example, a multi-taxon study showed that ten out of 15 taxa (including plants, invertebrates and vertebrates) did not show differences in richness between *Eucalyptus* monoculture and primary or secondary forest^9^.

Given the unclear influence of *Eucalyptus* plantations on conservation, it is necessary to assess biodiversity changes beyond the patterns of species richness, and to assess the plantations’ impact on ecological processes and species interactions. For instance, compared to native forest, tree monocultures have lower allocation and decomposition of organic matter^6^, different species interactions – promoting the outbreak of some species^10^ – and different community structure^11^. In this context, recent studies have shown that functional trait diversity is more strongly related to biotic interactions, abiotic filters, and ecological processes than taxonomic diversity^12,13^. Increasingly, researchers are assessing functional diversity through several indices: the dominant trait values, the occupied phenotypic space, the distribution of abundances in this space, or the ecological resilience and resistance to environmental disturbance or invasion of communities^14–17^. Although most of them are based on the average trait values of species, some methods have recently started to integrate intraspecific trait variation, which more accurately reflects ecosystem functioning and species responses to environmental changes^18–20^. Moreover, methods incorporating intraspecific variability give results that are less context-dependent, as well as more consistent with biologically expected patterns^21^.

In this study, we want to assess how *Eucalyptus* plantations affect the taxonomic and functional diversities of ant communities, considering intraspecific trait variation in the calculation of functional diversity. Ants are key in a myriad of essential ecological processes such as soil cycling and aeration, seed dispersion and decomposition of organic matter^22,23^. In addition, ants respond to environmental disturbances, may be used as a proxy for disturbance impact on others invertebrates^24^, making these insects good indicator of anthropic disturbances. Ant communities in tree plantation monocultures may have higher, lower, or equal richness comparing to native forest, but usually have different composition^11,25–28^. The lack of a clear pattern of how ant taxonomical diversity (richness) are affected by tree plantations and the change in species composition, along with the status of ants as important key organisms in several ecosystem functions, indicate that a functional trait approach may help to elucidate the effect of monoculture plantations on ant communities and its impacts on ecosystem. We sampled ants in managed *Eucalyptus* plantations with different management ages, as well as in unmanaged *Eucalyptus* plantations, and in native rain forest. We asked how *Eucalyptus* plantations affect the taxonomical and functional diversity indexes (alpha diversity) of these ant communities. We hypothesized that unmanaged plantations —where natural regeneration of the vegetation is starting to take place— would have higher functional and taxonomic diversities than managed ones, but lower diversities than the Atlantic Forest remnants. We also asked if *Eucalyptus* plantations influence the similarity (beta diversity) between ant communities, as an indication for possible effect on landscape homogenization. We expected that ant communities located in *Eucalyptus* plantations – regardless of age and management (managed or unmanaged) – would be more similar to each other than to forest ant communities (homogenization due to management).

## Results

**Figure 1:**
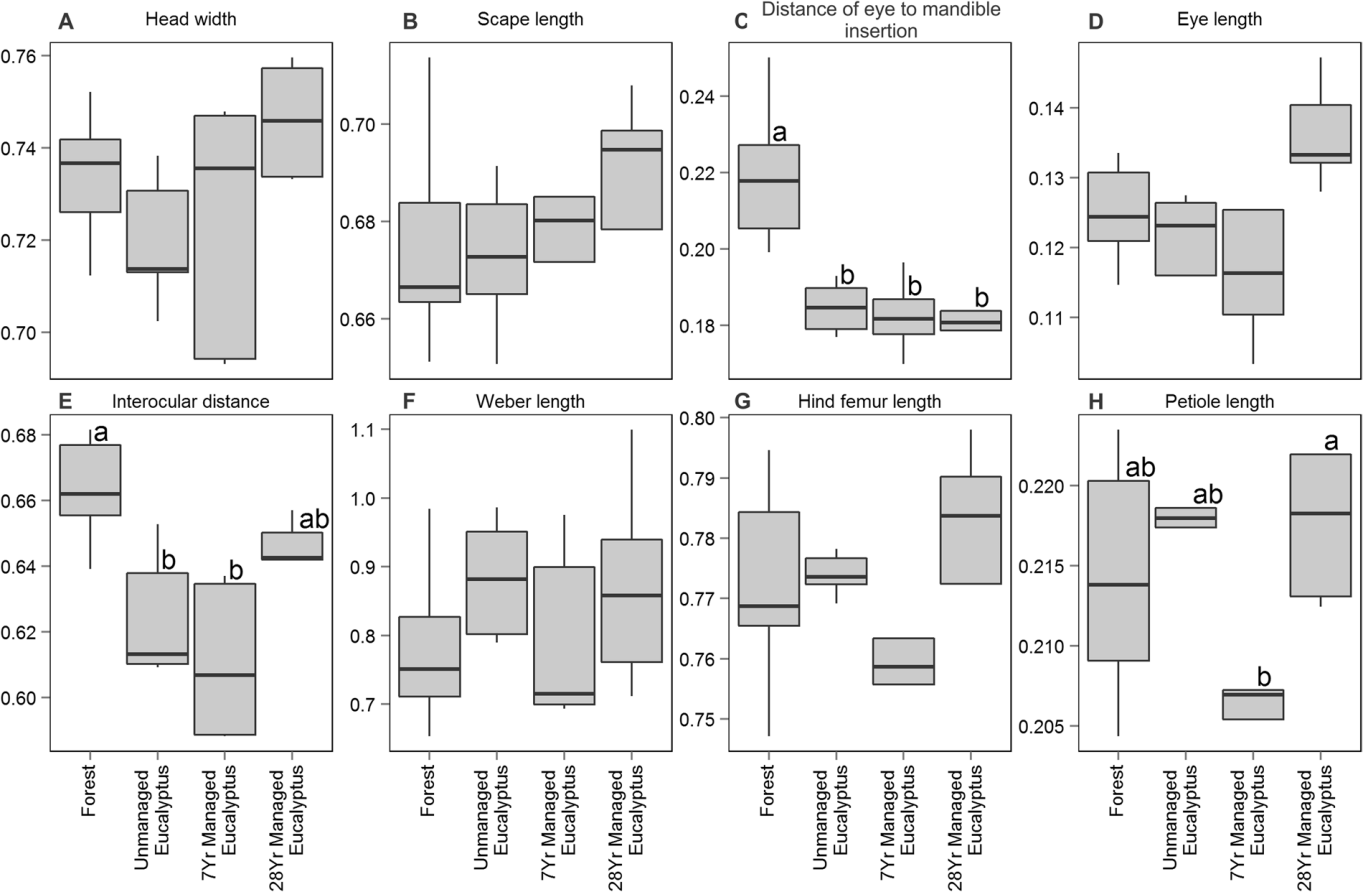
Mean and standard deviation of community weighted means of the eight functional traits of ant communities located in native rain forest, 28-year-old abandoned *Eucalyptus* plantations, seven-year-old commercial *Eucalyptus* plantations and 28-year-old *Eucalyptus* plantations. In each graphic, the different letters associated with the environments represent significant differences in the means assessed by Tukey’s Post-hoc analysis.

Three of the eight functional traits presented different community weighted mean (CWM) values between environments with different management (Fig. 1). Ant communities in rain forest had longer distance between the compound eye and mandible insertion (mean 0.210 mm ± SE 0.001) than those located in any of the eucalyptus plantations (0.180 mm ± 0.001, for both unmanaged *Eucalyptus*, 7-year-old *Eucalyptus* and 28-year-old *Eucalyptus*) (ANOVA: F = 11.58, p < 0.001) (Fig. 1B). Ant communities in the forest had longer interocular distances (i.e., eyes positioned more laterally) than ant communities located in unmanaged and 7-year-old *Eucalyptus* (0.662 mm ± 0.001 and 0.611 mm ± 0.001, respectively) (ANOVA: F = 11.0, p < 0.001) (Fig. 1E). For petiole length, differences were detected between communities of managed *Eucalyptus* of different ages, whereas communities in the older plantations had a smaller average petiole length (0.201 mm ± 0.001 and 0.223 mm ± 0.001, for 7-year-old *Eucalyptus* and 28-year-old *Eucalyptus*, respectively) (ANOVA: F = 3.09, p < 0.001) (Fig. 1H).

**Figure 2:**
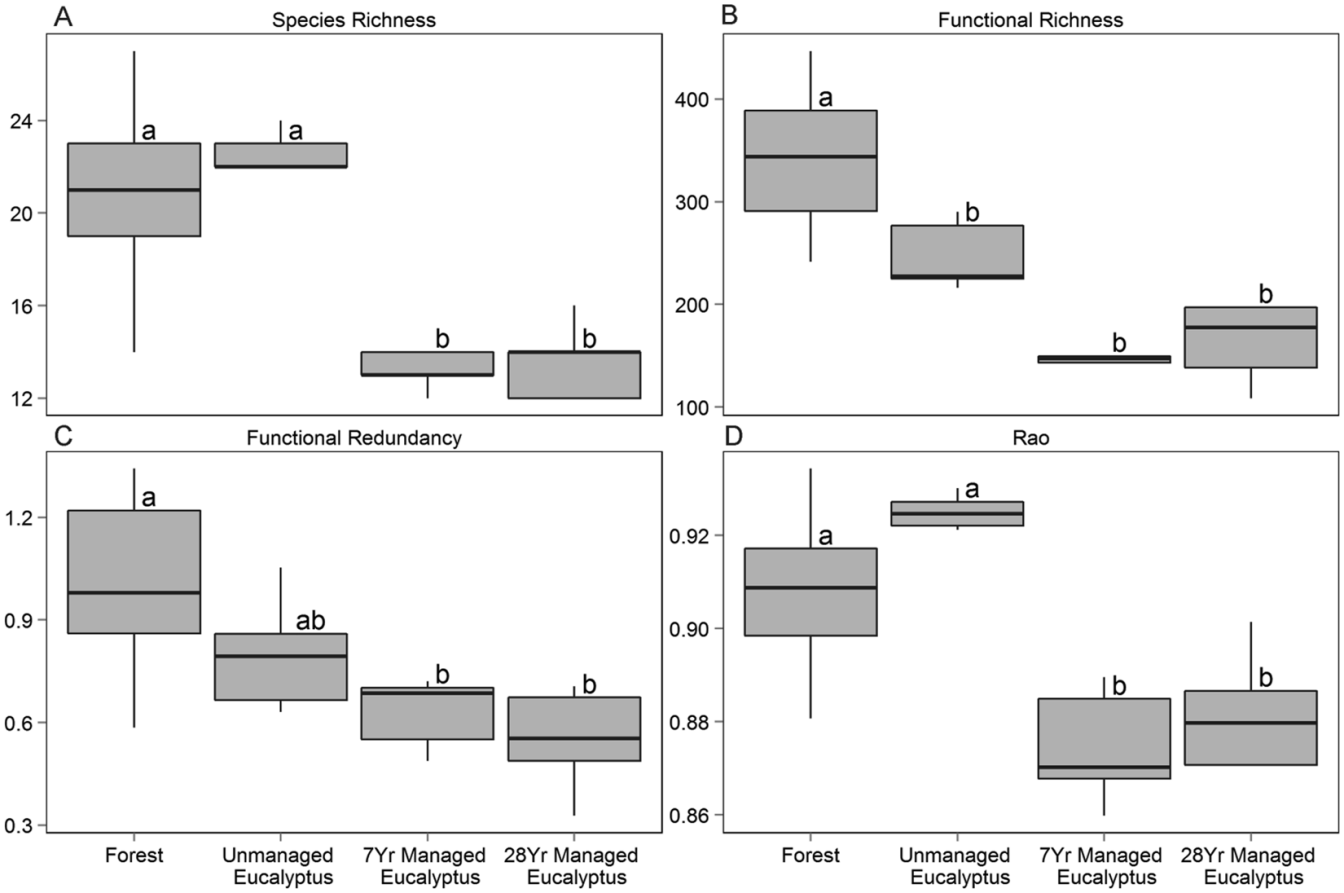
Mean and standard deviation of taxonomic (**A**) and functional (**B**–**D**) α diversity indices of ant communities located in native rain forest, 28-year-old unmanaged *Eucalyptus* plantations, seven-year-old commercial *Eucalyptus* plantations and 28-year-old *Eucalyptus* plantations. Different letters associated with the environments represent significant differences in the means assessed by Tukey’s Post-hoc analysis.

Managed *Eucalyptus* plantations had significantly decreased taxonomic and functional α diversities. Native rain forests and unmanaged *Eucalyptus* plantations had higher species richness (mean 20.701 ± SE 1.180 and 22.201 ± 0.660, respectively) than both recent and old *Eucalyptus* cultivations (13.8 ± 0.601 for 7-year-old *Eucalyptus* and 12.8 ± 1.352 for 28-year-old *Eucalyptus*) (Fig. 2A). Accordingly, native rain forests had the highest functional richness (FRic) values (308.461 ± 22.141), whereas the various types of *Eucalyptus* plantation did not differ in this aspect, regardless of their management regime (181.762 ± 17.562, 117.211 ± 25.584 and 139.684 ± 27.433, for unmanaged *Eucalyptus*, 7-year-old *Eucalyptus* and 28-year-old *Eucalyptus*, respectively) (Fig. 2B). We recorded the lowest values of functional redundancy (Fred) in managed *Eucalyptus* plantations (0.401 ± 0.041, 0.342 ± 0.043, for 7-year-old *Eucalyptus* and 28-year-old *Eucalyptus*, respectively); in contrast, native rain forests presented the highest values for this variable, while unmanaged plantations had intermediate values of functional redundancy (0.541 ± 0.081) (Fig. 2C). The results for Rao’s Q reflected the same pattern of species richness, with native rain forest and unmanaged *Eucalyptus* having higher values (0.920 ± 0.003 and 0.930 ± 0.001, respectively) than managed plantation (0.893 ± 0.005 and 0.891 ± 0.010, for 7-year-old *Eucalyptus* and 28-year-old *Eucalyptus*, respectively) (Fig. 2D).

**Figure 3:**
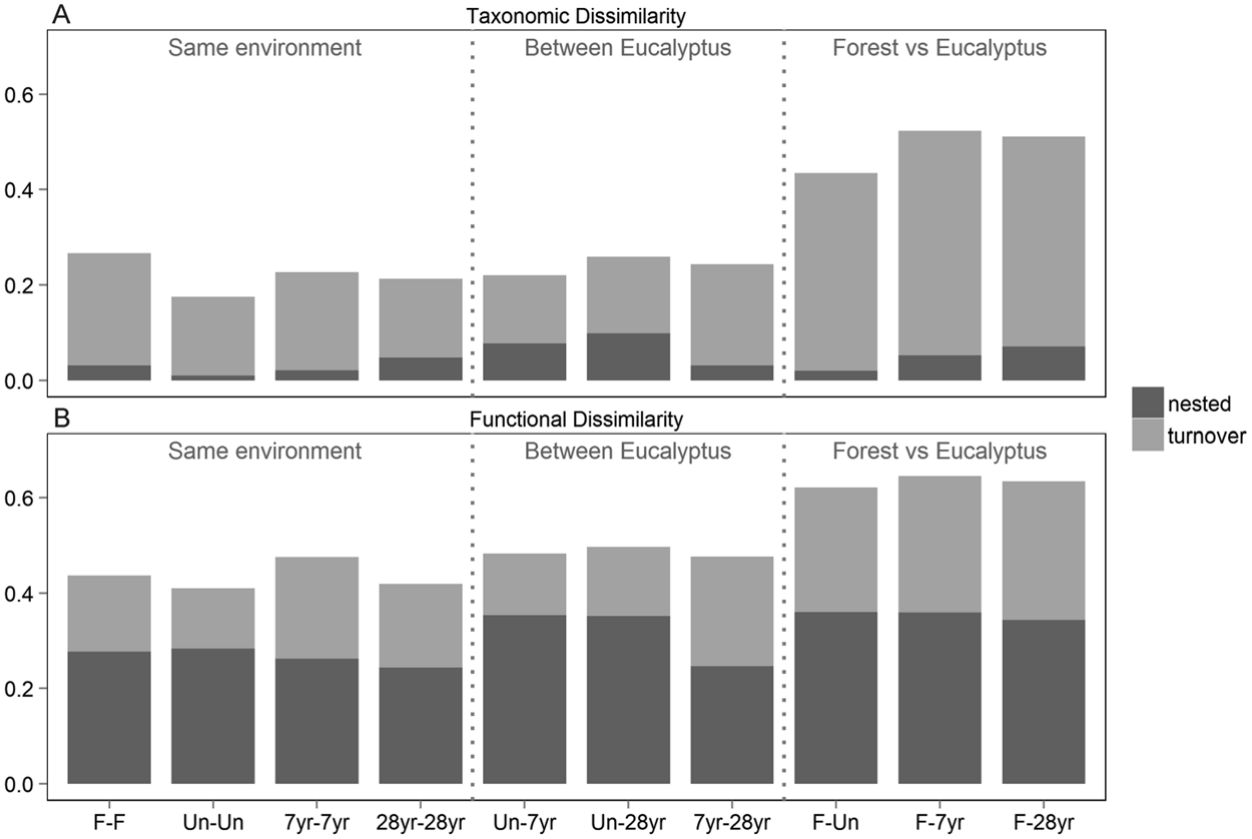
Mean of the total and the nested and turnover components of taxonomic (**A**) and functional (**B**) β diversities between environments (F = native rain forest; Un = 28-year-old unmanaged *Eucalyptus* plantations, 7 yr = recent seven-year-old *Eucalyptus* plantations; 28 yr = established 28-year-old *Eucalyptus* plantations).

PERMANOVA analyses revealed significant differences between environments in their taxonomic and functional β diversities, overall and for individual components (Table 1). Taxonomic and functional β diversities showed a similar pattern, revealing that the ant communities from native rain forest were markedly different from those of *Eucalyptus* environments, which were more similar among themselves (Fig. 3). In taxonomic β diversity, these high values were due mainly to the turnover in species composition (βsim), which accounted for most of the total β diversity (Fig. 3A). However, functional β diversity had no clear pattern: the highest dissimilarities—between native rain forest and *Eucalyptus* communities—received a similar contribution from both β diversity components (turnover and nestedness) (Fig. 3B).

**Table 1:** Results of PERMANOVA for taxonomic and functional β diversities for ant communities collected in forest fragments and managed and unmanaged *Eucalyptus* plantations of different ages, São Paulo State, Brazil. Individual components are presented.

*Β diversity*	*component*	*F*	*R²*	*p*
Taxonomic	All	17.900	0.719	0.001
	turnover	15.219	0.685	0.001
	nested	7.626	0.521	0.001
Functional	All	17.146	0.71	0.001
	turnover	2.867	0.29	0.001
	nested	2.026	0.224	0.001

## Discussion

*Eucalyptus* plantations are often thought to harbor a significant portion of native species and therefore have some value for ecosystem conservation^29,30^. Our results suggest that managed plantations greatly reduced both taxonomic and functional diversity of ant communities and further drove them to taxonomical and functional homogenization at the landscape level. However, unmanaged plantations emerged as a transition stage between managed plantations and native forests, showing α diversity values similar to those of native forests, while their taxonomic and functional compositions (β diversity) were more similar to those of managed *Eucalyptus* plantations. These results suggest that although indexes of taxonomical and functional diversity of ant communities in unmanaged *Eucalyptus* plantations could eventually approximate to those of communities in native forests in terms of α diversity, plantations have a long-term impact on community composition, making even abandoned areas more similar to managed conditions than unmanaged ones in terms of β diversity.

We found that ants in native rain forests had a longer distance from eyes to the mandible insertion compared to ants in *Eucalyptus* plantations. This trait is related to foraging activities, with predaceous ant species having higher values for this trait than non-predator ant species, *e.g*., Dacetini predators and specialist predators living in the soil^31^. This result suggests a decrease in the abundance and diversity of predator ants, which could be due to the fact that most of them feed on small invertebrates that compose the soil mesofauna^31,32^. Soil mesofauna is extremely sensitive to leaf litter structure, soil structure and composition^33^. According to Winck *et al*.^34^ *Eucalyptus* plantations can differ from native forest in many litter characteristics, such as moisture, mass and microbial activity, which can directly reduce mesofauna. Besides, the application of fertilizers and pesticides in *Eucalyptus* plantations, as well as the impoverishment of leaf litter and the high concentration of allelopathic substances that characterize these monocultures^35^ might affect the availability of this resource for ants. This result concurs with the notion that ants are the best indicator of invertebrate assemblage composition in disturbed environments^36,37^. Moreover, the simplification of the vegetation structure and the leaf litter characteristics in *Eucalyptus* monocultures can also substantially reduce the diversity of arthropods and hinder the coexistence of ant species^38,39^. More complex environments (in this study, native rain forest) provide a myriad of microhabitats, many of which are not accessible to dominant species, and thereby allow the existence of other species and facilitate coexistence in ant communities^39^.

Compared to unmanaged *Eucalyptus* plantations, managed plantations had a more negative effect on the taxonomic and functional diversity of ant communities, as reflected by their low values on all α-diversity indices. Managed *Eucalyptus* plantations had approximately half of the ant species that were found in the native rain forest, corresponding to a similar decrease in functional richness. At the same time, functional redundancy in managed plantations was lower than in the native rain forest, suggesting that the functional diversity of ant communities in plantations might be less resistant to species loss^40–43^. Ant communities located in highly disturbed environments and in non-native vegetation generally have a reduced number of species, a few of which are dominant^44,45^. Such species, *e.g*. species from Dominant Dolichoderinae and Generalist Myrmicinae functional groups^46^, are better adapted to the disturbed conditions and have rapid recruitment to find and defend food resources, driving competing species to local extinction^36,47,48^. More than reducing species richness and the amount of functional trait space occupied by ant communities, managed *Eucalyptus* plantations also decreased the functional similarities between species (functional redundancy), likely reducing community resistance. This interpretation is also supported by the low values of Rao’s Q in communities located in managed *Eucalyptus*, indicating the low evenness of functional diversity in these communities. In summary, altered environmental conditions associated with managed plantations benefited only a few species, resulting in a functional homogenization of ant communities, with higher similarity among species (reflected by lower Rao’sQ values) as well as a higher vulnerability (reflected by lower functional redundancy values).

Functional diversity of ant communities showed that unmanaged *Eucalyptus* plantations performed as a transition stage between communities located in managed plantations and native rain forest: they had species richness and Rao’s Q values similar to those of communities located in native rain forest, but they occupied a smaller functional space (functional richness). In studies performed in secondary forest derived from pastures abandoned up to 50 years, ant richness did not reach the values of communities located in native old grow forest^49^. The authors hypothesized that the recovery of ant communities established in former pasture would be slower compared to areas where the land is less disturbed. In line with such claim, our results suggest that –although *Eucalyptus* plantations cause severe impacts on the environment– its forest phytophisionomy may provide resources that allow ant communities to recover richness faster than in other land uses. On the other hand, the reduction in functional richness may result from the absence of certain resources in the unmanaged *Eucalyptus*, because the recovery of species composition can take even longer –although the recovery of plant community richness in tropical forest can take up to 40 years^50,51^. Furthermore, *Eucalyptus* trees may inhibit the development of some plant species by allelopathy, reducing the complexity of the environment^52^, as well as the availability of resources for ants, thereby influencing the amount of functional space occupied by ant communities^52,53^. Reduction of functional richness was also found for dung beetles –other insects group frequently used as bioindicator of human impact on biodiversity– after restoration of native Atlantic Forest from *Eucalyptus* plantation^54^. According to these authors, such result may indicate a deficiency in ecosystem processes supported by these organisms, as well as a reduction in the temporal stability of these systems.

Indicators of diversity at the local level (α diversity) of ant communities from unmanaged *Eucalyptus* plantations resembled those located in native forest, which suggests that the natural regeneration of forests in unmanaged *Eucalyptus* plantations can attenuate the dominance of some species in managed plantations. However, ant communities in unmanaged *Eucalyptus* plantations were taxonomically and functionally more similar to the managed plantations, as revealed by the patterns of β diversity. This result highlights that the consideration of different aspects of diversity is important for understanding the impacts of human disturbance on ecosystems^53,55^. Biologically, our results reveal that, for managed *Eucalyptus* plantation, 28 years of abandonment were not enough for the ant communities to recover the characteristics of those found in native forests. Studies with others organism, such as dung beetles^54,56^, lizards^57^, or arachnids^58^, also suggest that communities in native forest differ not just from plantations forests, but also from secondary or restored forests, demonstrating the uniqueness of primary forests and the importance of maintaining these habitats.

Notwithstanding the equal power of nestedness and turnover components to explain functional β diversity, the latter was the main driver of dissimilarities in taxonomic β diversity, exposing great changes in species composition. Our results agree with Bihn *et al*.^59^, who suggested that the high functional diversity of ant communities in tropical forest is driven primarily by rare species, which are often more sensitive to habitat disturbance. Consequently, the taxonomic and functional structure of these communities can only be attained in primary forests. Lapola & Fowler^60^ also found that ant communities in forest fragments shared more similarities among themselves than with ant communities located in 100-year-old *Eucalyptus* plantations established for restoration purposes, which supports the idea that *Eucalyptus* plantations have a long-lasting impact on ant communities.

We highlight that management (or lack thereof) is a major driver of the differences in the functional structure of ant communities in *Eucalyptus* plantations. The process of natural forest regeneration, known as “passive restoration”, is currently suggested as one of the most promising methods for ecosystem restoration, because it is cheaper than the artificial tree plantation (i.e. active restoration)^61^. Natural forest regeneration allows natural colonization and secondary succession by any organisms that can disperse and establish in abandoned agricultural areas^61^. Although passive restoration can be faster and as effective as active restoration, especially in tropical and humid temperate ecosystems, it is highly stochastic and dependent on organisms’ dispersal abilities as well as on biotic and abiotic conditions^61–63^. In this sense, cessation of logging activities in *Eucalyptus* plantations may accelerate the restoration process, because of the positive microclimatic effect of the plantation canopy and the attraction of seed-dispersing fauna^36,64^.

In this study, we found that ant communities in abandoned *Eucalyptus* plantations approximated the functional structure of communities in native rain forest. This functional restructuring of the ant community in abandoned *Eucalyptus* plantation, comparing to those in managed *Eucalyptus* plantation, is probably a consequence of changes in trophic interactions involving ants. Most importantly, considering the key role of ants in ecological processes such as soil cycling, seed harvesting and seed dispersion^22,23^, this restructuring might further accelerate the recovery of the conditions of the whole system. In this sense, the implementation of restoration strategies, such as the applied nucleation of native vegetation patches in unmanaged *Eucalyptus* plantation, may increase the diversity of resources for ant communities, consequently increasing its functional richness as well as its importance in ecological and restoration processes^65^.

Our findings support the idea that *Eucalyptus* plantations can severely affect the taxonomic and functional diversities of ant communities, and that these impacts have long-term effects, even in unmanaged plantations. On the other hand, the passive restoration of native rain forest in unmanaged plantations can change the functional structure of ant communities, increasing their functional diversity and redundancy tending to converge with that of native rain forests’ communities. Although it is impracticable, particularly for economic reasons, to recommend the abandonment of monoculture tree plantations or the cessation of their management as a conservation strategy^29^, our outcomes suggest that the implementation of restoration strategies, such as passive restoration between stands of tree plantation, may mitigate some impacts of this land use on ant communities and on the various ecological processes in which theses insects are essential.

## Methods

### Study area & sampling design

The study was conducted at the Alto Tietê and Itatinga River basins, in São Paulo, Brazil. The landscape comprises a mosaic of agriculture, urban areas, *Eucalyptus* plantations under different management regimes, and native rain forest remnants. Even though *Eucalyptus* plantations are usually logged every seven years, management practices vary across the studied area, resulting in a mosaic of plantations of different ages. The native rain forest remnants in this region are categorized as Ombrophilous Dense Forest, which is characteristic of high-precipitation regions^66^. These remnants are composed of evergreen phanerophytes, with an average height of 15 m, and a dense shrub vegetation of palms, lianas, epiphytes and ferns.

We selected 25 sites distributed throughout four types of forest stand: (a) recent *Eucalyptus saligna* monoculture, seven years old and uncut (n = 5); (b) established *E. saligna* monoculture, 28years old and clearcut 3 times (n = 5); (c) abandoned *E. saligna* plantation, left without management for 28 years prior to this study (n = 5); and (d) original native rain forest (n = 10). The distances between plots of the same treatment ranged from a minimum of 264 m (native Atlantic forest) to a maximum of 1.8 km (28-year-old *Eucalyptus* monoculture).

The short distance between some of the plots was a due to geographical and sampling constrains in the sampled landscape, since we made a thorough effort in order to homogenize the characteristics of the sites by excluding confounding or hidden effects. To account for spatial auto-correlation we performed two spatial auto-correlation tests. First, to assess whether there was spatial autocorrelation between the geographic coordinates’ dissimilarity matrix and each response variable’s dissimilarity matrix, we calculated Moran’s I values, followed by a Monte Carlo permutation (N = 9,999). As non-significant spatial autocorrelation was detected for all dependent variables (Supplementary Table S4 in Supplementary Information), we considered the spatial sampling effect to be irrelevant. Second, to assess whether there was spatial autocorrelation in communities’ composition, we performed a spatial auto-correlation analysis between community composition (Bray-Curtis distance) and the spatial distance between samples, which showed a poor autocorrelation (Mantel r = 0.28 [which corresponds to only a r^2^ = 0.08], in Figure S5 in Supplementary Material).

In each site, we established a 250-m transect in which we systematically established six sampling sites at 50-m intervals. In neighboring sites where the distance was small, we oriented the transects in order to maintain the greatest possible distance between sites. In each of the six sampling site of each transects, we collected 0.5 m² of leaf litter and transferred it to a Berlese funnel, where it remained for seven days to sample ants. During that time, the ants dropped out of the Berlese funnel mesh sack, and we collected them live in plastic cups with moist sponges that were later transferred to vials containing 80% ethanol^67^. All specimens were identified to morphospecies or species level, whenever possible, and deposited in the Myrmecological Laboratory, University of Mogi das Cruzes. Each site was sampled once during the rainy season (the period of higher ant activity) between September and December of the years 2010 and 2011.

### Functional traits

We measured functional individual-level traits, selecting up to six individuals of each species (or morphospecies) in each sampling site, depending on their abundance. Using this procedure we guaranteed, especially for more common species, a robust trait sampling, in a way that for more than half of species (43 of 78 species) we measured more than six individuals (Table S1 and Figures S1 and S2 in Supplementary Information). Six individuals are the most frequently used number of individuals measured in studies of functional diversity of ants^68–72^. The number of individuals reached up to 104 individuals measured for one species (*Solenopsis* sp2). For some species, we had a more limited number of individuals, but these species were generally rarer, and accounted for a very low percentage of individuals in the communities (Figure S3 in Supplementary Information). This lack of information is expected not to affect significantly the results, as shown repeatedly in the several studies: Pakeman & Quested^73^ Pakeman^74^ and Majekova^75^, which also considered functional diversity of ants.

To calculate functional diversity indices, we selected eight morphological traits related to the species’ ecological roles^70,76^: head width, scape length, distance of eye to the mandible insertion, eye length, minimum inter-eye distance, Weber’s length, petiole height (disregarding the postpetiole) and hind leg length (sum of tibia and femur) (Table 2). We standardized all others traits, dividing each trait by Weber’s length to reduce correlation with body size. We subsequently log-transformed trait values to reduce the influence of extreme values, and standardized the values to have mean of 0 and unit variance. Then, we applied such procedures to be able to apply (Principal Component Analysis; PCA) in a further step (see below), since PCA assumes that there is a linear relation between traits, which can be skewed by extreme values.

**Table 2:** Morphological traits used to calculate functional diversity and its functional significance for ant communities sampled in 25 sites within Atlantic Forest patches and *Eucalyptus* plantations of varying ages and management strategies, São Paulo State, Brazil.

*Trait*	*Functional significance*
Head width	Indicative of the size of spaces through which ant can pass^67^ and mandibular musculature (wider heads accommodate larger mandibular muscles that therefore allow capture of larger or fiercer prey)^94^
Scape Length	Indicative of sensory abilities (longer scapes facilitates the following of pheromone trails)^95^
Distance of eye to the mandible insertion	Indicative of behavior and visual performance of ant species^96,97^
Eye length	Indicative of foraging period, food-searching behavior^95^ and habitat type^98^
Interocular distance	Perception of habitat complexity^71^ and the performance of visual predators^96^; in general, predatory ant species have compound eyes set farther apart on the head capsule than in other species^31^
Weber’s length	Indicative of body size, which is related to prey size selection during solitary foraging^99^, microhabitats in which different species forage^69,95^, metabolic characteristics^72^ and many life history traits such as resource use^100,101^
Leg length (femur + tibia)	Indicative of complexity of the habitat occupied^69,102^, thermal and dry resistance (^103^, locomotion abilities^102^ and food-searching behavior^95^.
Petiole length (not including postpetiole, if present)	Correlated to behavior of predator species and performance in prey capture^68^

### Taxonomic diversity

We calculated α taxonomic diversity of the ant community (species richness) at each site. To assess the taxonomic diversity between communities, we computed β taxonomic diversity for all possible pairs of ant communities, as proposed by Baselga^77^. This procedure consists of estimating total β diversity using the Sorensen dissimilarity index (βsor), then decomposing it into two components: turnover β diversity, which is estimated through the Simpson dissimilarity index (βsim), and nested β diversity, which is the difference between βsor and βsim (βnes). Turnover and nested β diversities reflect species replacement and the loss of species between sites, respectively, and they reveal the process involved in community assembly^77^.

Taxonomic β diversity was calculated using the function beta.pair from the betapart package^78^ in R software^79^.

### Functional diversity

We calculated the community-weighted mean (CWM) of each functional trait, using the mean trait value of each species, weighted according to its local abundance; CWM reflects the dominant trait value of the community^80^. To calculate the other functional diversity indexes, we first performed a Principal Component Analysis (PCA) using the eight traits as input. It allowed us to synthesize the major axes of variation in the functional space and reduce the number of dimensions used to calculate the functional diversity indices^81,82^. We performed the PCA analysis using the mean trait values of each species and, afterwards, we predicted the values of PCA axes for all measured individuals. We adopted this procedure (using mean value of traits of each species to perform the PCA) because the use of values from individuals to performed the PCA analysis might biased the PCA axes due to species with high number of measured individuals. We retained the first four axes of this PCA, which accounted for 86.4% of the total variance in traits, and performed all subsequent analyses using those axes as indicators of the functional space.

For each ant community, we estimated functional richness (FRic), functional redundancy (FRed) and Rao’s Q. FRic represents the amount of functional trait space occupied by the community^16^. FRed is the saturation of the functional space of the community and reflects the potential resilience and resistance of the community^20,40,83^. Rao’s Q expresses the pairwise functional differences between species of the community, weighted by their relative abundances^21,84^. For Rao’s Q we used of species occurrence on each site instead of species abundance, because abundance may be biased by the number of individuals inhabiting the colony^85,86^.

Finally, we calculated functional β diversity, which expresses the functional dissimilarities between all possible pairs of ant communities. We further decomposed this index into the turnover and nested components, representing the replacement of functional traits and the loss of common functional traits among communities, respectively^20,87,88^.

CWM was calculated using the “dbFD” function in the “FD” package (^89^, and PCA ordination was calculated using the “prcomp” function; both calculations were run in R software^79^. All functional diversity indices were calculated using Trait Probability Distribution (TPD), which incorporates intra-specific variation, the multidimensional nature of traits, species abundances and probabilistic trait distributions^20^. The first step to calculate functional diversity with the TPD approach is to compute the Trait Probability Distribution of each species (TPDs), which reflects the probability of observing different trait values in a given species. This approach requires the construction of a multidimensional probability density function using the individuals of each species. We opted to estimate these distributions using a multivariate Gaussian distribution, which requires the average value of each species in the multidimensional trait space as well as their standard deviation (reflecting the variability in trait values among conspecifics) for each axis. Therefore, we included intraspecific variation in functional diversity indices by using the standard deviations of each species. We used two different strategies to estimate standard deviations, depending on the number of measured individuals of each species. For species with at least six measured individuals (total of 46 species), we estimated standard deviations based on the data from all the individuals of that species in the entire sample. For species with less than six measured individuals (33 species), we used the average standard deviation of all the other species as their standard deviation. This procedure retains the standard deviation of the most abundant species, while assigning a reasonable standard deviation value to the least sampled species, which is preferable to other alternatives, such as using the same standard deviation (SD) value for all species^89^.

In the TPD framework, the TPDs are further used to estimate the trait probability distribution of each community (TPDc), which is the sum of the TPDs of all species in the community, weighted by their relative abundances (which we estimated using frequencies of occurrence)^20^.

### Data analysis

To assess the effect of *Eucalyptus* management on taxonomic and functional ant diversity, we first conducted a one-way-ANOVA followed by a Tukey’s post hoc analysis for α taxonomic and functional diversity indices (richness, FRic, FRed and Rao’s Q). However, in the case of Rao’s Q, which presented non-normal residuals (Shapiro-Wilk test), we used the Kruskal-Wallis test followed by a non-parametric multiple comparison test^90^. To assess the effect of managed *Eucalyptus* on taxonomic and functional β diversities, we used PERMANOVA (9,999 permutations)^91^ for total β diversity and its components, turnover and nestedness, between sites.

The distance matrix used to calculate the Moran’s I values was computed using the vegan^92^ package in R software, and all graphics were produced using the ggplot2^93^ package in R software^79^.

### Data Availability

The datasets generated and analyzed during the current study are available from the corresponding author on reasonable request.

## Electronic supplementary material

Supplementary information
