# Supplementary material for: Homogenization and impoverishment of taxonomic and functional diversity of ants in Eucalyptus plantations
Source: Sci Rep. 2018 Feb 19;8:3266. doi: 10.1038/s41598-018-20823-1 (PMC5818526; doi:10.1038/s41598-018-20823-1)
Supplement: Supplementary file 1 — Supplementary information [file 41598_2018_20823_MOESM1_ESM.pdf]

Homogenization and impoverishment of taxonomic and functional diversity of ants in *Eucalyptus* plantations

Martello, Felipe; de Bello, Francesco; Morini, Maria Santina de Castro; Silva, Rogério R.; Souza-Campana, Débora Rodriges; Ribeiro, Milton Cezar; Carmona, Carlos Pérez.

Supplementary Information

Supplementary Table S1. Number of individuals measured (N), means and standard deviation (SD) of the eight traits used.

Species	***N***	***Weber's length***		***Head width***		***Interocular distance***		***Leg length***		***Scape length***		***Distance of eye to mandible insertion***		***Eye length***		***Petiole length***	
		Mean	*SD*	*Mean*	*SD*	*Mean*	*SD*	*Mean*	*SD*	*Mean*	*SD*	*Mean*	*SD*	*Mean*	*SD*	*Mean*	*SD*
Acanthognathus ocellatus	3	0.925	0.039	0.771	0.020	0.537	0.023	0.900	0.026	0.839	0.052	0.386	0.079	0.162	0.014	0.286	0.026
Acantognathus rudis	1	0.843	-	0.794	-	0.647	-	1.059	-	1.000	-	0.382	-	0.265	-	0.294	-
Acromyrmex diasi	2	2.239	0.124	0.775	0.029	0.638	0.022	1.174	0.074	0.767	0.070	0.186	0.004	0.137	0.008	0.167	0.023
Acromyrmex disciger	6	1.485	0.464	0.804	0.044	0.683	0.038	1.070	0.093	0.809	0.096	0.152	0.034	0.128	0.009	0.152	0.023
Acromyrmex rugosus rochai	10	1.421	0.214	0.769	0.043	0.623	0.042	1.015	0.156	0.704	0.056	0.147	0.017	0.087	0.011	0.156	0.022
Apterostigma sp1	3	1.290	0.025	0.552	0.021	0.506	0.015	0.878	0.011	0.545	0.019	0.270	0.041	0.109	0.020	0.205	0.036
Apterostigma sp2	7	1.516	0.107	0.526	0.044	0.469	0.041	0.920	0.066	0.625	0.059	0.230	0.015	0.131	0.009	0.173	0.015
Basiceros rugiferum	45	0.558	0.034	0.884	0.139	0.911	0.075	0.823	0.109	0.569	0.104	0.466	0.081	0.112	0.025	0.251	0.032
Basiceros stenognathum	16	0.522	0.037	0.908	0.193	0.764	0.065	0.777	0.082	0.581	0.067	0.508	0.086	0.093	0.012	0.271	0.032
Brachymyrmex admotus	56	0.532	0.034	0.862	0.046	0.739	0.056	0.970	0.088	0.997	0.100	0.228	0.023	0.218	0.020	0.116	0.016
Brachymyrmex cordemoyi	1	0.338	-	0.941	-	0.765	-	1.000	-	0.941	-	0.294	-	0.176	-	0.118	-
Brachymyrmex heeri	27	0.406	0.052	0.951	0.151	0.767	0.097	0.951	0.102	0.939	0.091	0.206	0.064	0.195	0.028	0.129	0.056
Camponotus sp8	1	1.679	-	0.593	-	0.444	-	0.759	-	0.759	-	0.278	-	0.222	-	0.130	-
Carebara sp1	1	0.320	-	0.654	-	0.885	-	0.654	-	0.500	-	0.231	-	0.077	-	0.231	-
Cerapachys splendens	3	0.877	0.271	0.692	0.117	0.637	0.100	0.660	0.151	0.404	0.073	0.298	0.134	0.108	0.067	0.469	0.078
Crematogaster corticicola	5	0.883	0.041	0.878	0.055	0.889	0.052	1.022	0.056	0.799	0.047	0.327	0.027	0.225	0.011	0.263	0.054
Crematogaster sp1	13	0.677	0.047	0.779	0.061	0.774	0.043	0.899	0.070	0.828	0.045	0.340	0.027	0.198	0.021	0.302	0.082
Crematogaster sp7	7	0.588	0.107	0.877	0.119	0.777	0.098	0.862	0.082	0.723	0.074	0.347	0.055	0.277	0.044	0.389	0.061
Cyphomyrmex gr rimosus	54	0.847	0.077	0.723	0.048	0.580	0.051	0.876	0.101	0.595	0.052	0.226	0.034	0.177	0.020	0.174	0.020
Cyphomyrmex sp1	3	1.017	0.043	0.765	0.029	0.627	0.090	0.748	0.036	0.389	0.099	0.163	0.019	0.122	0.005	0.195	0.008

Cyphomyrmex transversus	12	0.876	0.089	0.761	0.039	0.599	0.052	0.929	0.089	0.615	0.054	0.201	0.017	0.160	0.014	0.153	0.019
Dyscothyrea sexarticulata	4	0.453	0.019	0.836	0.098	1.021	0.506	0.959	0.816	0.460	0.039	0.435	0.347	0.101	0.115	0.220	0.009
Ectatomma edendatum	14	2.882	0.086	0.530	0.031	0.459	0.028	0.853	0.027	0.626	0.031	0.247	0.015	0.137	0.004	0.248	0.044
Gnamptogenys continua	3	1.017	0.066	0.564	0.106	0.531	0.098	0.528	0.029	0.465	0.036	0.236	0.026	0.049	0.003	0.382	0.022
Gnamptogenys striatula	80	1.313	0.100	0.663	0.047	0.593	0.035	0.868	0.077	0.734	0.063	0.293	0.023	0.123	0.011	0.288	0.027
Heteroponera dentinodis	1	1.166	-	0.809	-	0.638	-	0.638	-	0.468	-	0.298	-	0.170	-	0.340	-
Heteroponera mayri	12	0.986	0.054	0.684	0.038	0.596	0.027	0.600	0.037	0.487	0.029	0.289	0.043	0.189	0.011	0.298	0.023
Hylomyrma balzani	10	0.866	0.087	0.809	0.056	0.716	0.076	0.721	0.070	0.563	0.060	0.139	0.023	0.171	0.042	0.313	0.035
Hylomyrma reitteri	25	1.117	0.051	0.862	0.043	0.772	0.047	0.738	0.057	0.516	0.036	0.139	0.007	0.195	0.009	0.336	0.017
Hypoponera sp1	33	0.808	0.064	0.675	0.038	0.576	0.037	0.659	0.050	0.580	0.045	0.093	0.007	0.046	0.014	0.209	0.021
Hypoponera sp10	1	1.782	-	0.639	-	0.528	-	0.639	-	0.528	-	0.111	-	0.056	-	0.194	-
Hypoponera sp11	4	1.184	0.071	0.639	0.030	0.575	0.037	0.597	0.009	0.598	0.034	0.153	0.031	0.047	0.007	0.193	0.025
Hypoponera sp4	26	0.846	0.075	0.655	0.069	0.583	0.072	0.662	0.075	0.593	0.083	0.118	0.012	0.030	0.003	0.209	0.021
Hypoponera sp5	1	0.698	-	0.578	-	0.511	-	0.400	-	0.400	-	0.089	-	0.044	-	0.200	-
Hypoponera sp7	15	1.213	0.093	0.601	0.048	0.521	0.040	0.594	0.058	0.538	0.047	0.113	0.007	0.042	0.004	0.213	0.024
Hypoponera sp8	48	0.999	0.090	0.680	0.041	0.585	0.033	0.685	0.057	0.637	0.052	0.162	0.034	0.046	0.010	0.223	0.028
Lachnomyrmex plaumani	4	0.732	0.032	0.873	0.029	0.703	0.011	0.679	0.030	0.493	0.035	0.136	0.006	0.238	0.010	0.212	0.033
Linepithema neotropicum	31	0.785	0.105	0.756	0.064	0.426	0.064	0.834	0.091	0.762	0.088	0.156	0.036	0.162	0.020	0.098	0.017
Megalomyrmex goeldii	1	1.240	-	0.581	-	0.484	-	0.944	-	0.839	-	0.177	-	0.137	-	0.202	-
Megalomyrmex iheringi	9	1.702	0.112	0.575	0.050	0.464	0.025	0.986	0.083	0.849	0.086	0.166	0.011	0.142	0.009	0.205	0.022
Monomorium pharaonis	1	0.595	-	0.708	-	0.583	-	0.583	-	0.750	-	0.208	-	0.083	-	0.208	-
Mycetarotes senticosus	2	1.159	0.149	0.647	0.000	0.596	0.010	0.958	0.080	0.885	0.059	0.240	0.049	0.118	0.000	0.152	0.007
Myrmelachista catharinae	5	0.601	0.052	0.809	0.052	0.718	0.043	0.745	0.110	0.565	0.054	0.300	0.025	0.167	0.014	0.173	0.023
Nylanderia sp1	16	0.740	0.041	0.668	0.037	0.462	0.065	0.892	0.057	0.935	0.043	0.214	0.033	0.169	0.014	0.155	0.024
Odontomachus affinis	1	4.350	-	0.545	-	0.418	-	0.855	-	0.763	-	0.126	-	0.126	-	0.163	-
Odontomachus meinerti	8	2.444	0.053	0.659	0.039	0.520	0.035	0.866	0.052	0.798	0.033	0.203	0.004	0.162	0.004	0.208	0.017
Pachycondyla harpax	4	1.910	0.146	0.679	0.014	0.581	0.031	0.679	0.014	0.528	0.018	0.135	0.047	0.115	0.009	0.253	0.124
Pachycondyla sp2	10	1.523	0.066	0.613	0.021	0.510	0.022	0.712	0.040	0.655	0.027	0.163	0.007	0.082	0.004	0.196	0.009
Pachycondyla striata	9	4.456	0.227	0.667	0.029	0.535	0.022	0.750	0.063	0.604	0.030	0.087	0.034	0.170	0.016	0.290	0.021
Paratrechina longicornis	48	0.725	0.066	0.667	0.047	0.434	0.037	0.920	0.109	0.911	0.105	0.207	0.017	0.172	0.014	0.141	0.015

Pheidole cf dione	1	0.746	-	0.833	-	0.750	-	0.750	-	0.625	-	0.167	-	0.125	-	0.167	-
Pheidole gertrudae	8	0.967	0.042	0.747	0.036	0.695	0.019	0.994	0.030	0.882	0.051	0.205	0.027	0.170	0.013	0.141	0.020
Pheidole pr senilis	48	1.033	0.068	0.593	0.029	0.571	0.026	0.872	0.043	0.801	0.040	0.156	0.020	0.129	0.011	0.131	0.015
Pheidole sospes	100	0.483	0.038	0.758	0.100	0.805	0.069	0.829	0.103	0.777	0.091	0.174	0.041	0.162	0.022	0.181	0.023
Pheidole sp12	18	1.210	0.066	0.651	0.030	0.654	0.037	0.878	0.030	0.783	0.048	0.179	0.019	0.123	0.009	0.121	0.014
Pheidole sp14	6	0.859	0.019	0.679	0.026	0.646	0.021	0.814	0.059	0.670	0.042	0.173	0.003	0.116	0.002	0.135	0.016
Pheidole sp15	1	0.716	-	0.833	-	0.778	-	0.778	-	0.639	-	0.167	-	0.111	-	0.167	-
Pheidole sp16	27	0.547	0.050	0.837	0.052	0.776	0.034	0.717	0.053	0.725	0.051	0.171	0.028	0.125	0.019	0.169	0.024
Pheidole sp18	7	0.724	0.049	0.812	0.045	0.765	0.036	0.734	0.030	0.676	0.043	0.168	0.024	0.143	0.014	0.167	0.014
Pheidole sp20	2	0.818	0.035	0.698	0.030	0.621	0.005	0.939	0.003	0.879	0.005	0.152	0.006	0.152	0.006	0.152	0.006
Pheidole sp28	18	0.589	0.114	0.835	0.099	0.749	0.097	0.782	0.130	0.728	0.104	0.124	0.035	0.168	0.027	0.153	0.025
Pheidole sp29	10	0.849	0.019	0.651	0.048	0.624	0.052	0.990	0.058	0.914	0.034	0.173	0.028	0.156	0.021	0.140	0.016
Pheidole sp31	5	0.992	0.000	0.635	0.029	0.540	0.014	0.685	0.089	0.685	0.034	0.150	0.000	0.175	0.000	0.200	0.000
Pheidole sp36	7	0.847	0.046	0.710	0.044	0.652	0.031	1.018	0.048	0.922	0.040	0.206	0.012	0.176	0.011	0.167	0.011
Pheidole sp43	16	0.797	0.143	0.760	0.058	0.710	0.049	0.778	0.088	0.695	0.062	0.179	0.041	0.125	0.013	0.134	0.020
Pheidole sp9	2	0.886	0.066	0.756	0.031	0.667	0.000	0.880	0.065	0.863	0.089	0.176	0.013	0.176	0.013	0.176	0.013
Pheidole triconstricta	4	0.818	0.035	0.728	0.026	0.584	0.029	0.888	0.090	0.865	0.034	0.152	0.007	0.152	0.007	0.136	0.016
Pyramyca sp9	5	0.827	0.064	0.677	0.030	0.521	0.044	0.694	0.050	0.416	0.048	0.264	0.019	0.113	0.008	0.271	0.009
Solenopsis sp2	104	0.411	0.023	0.785	0.052	0.711	0.049	0.622	0.077	0.601	0.063	0.130	0.034	0.082	0.014	0.244	0.025
Solenopsis sp3	84	0.473	0.080	0.771	0.061	0.677	0.054	0.635	0.062	0.599	0.060	0.139	0.036	0.088	0.025	0.230	0.035
Solenopsis sp4	22	0.413	0.037	0.794	0.061	0.717	0.058	0.654	0.080	0.624	0.063	0.128	0.028	0.088	0.008	0.239	0.037
Strumigenys appretiata	5	0.579	0.012	0.818	0.093	0.610	0.147	0.731	0.055	0.426	0.144	0.375	0.100	0.097	0.011	0.248	0.014
Strumigenys cosmostela	7	0.627	0.028	0.735	0.032	0.509	0.020	0.810	0.053	0.620	0.099	0.396	0.017	0.079	0.003	0.238	0.010
Strumigenys crassicornis	26	0.516	0.046	0.572	0.052	0.483	0.076	0.731	0.066	0.515	0.091	0.417	0.072	0.090	0.014	0.222	0.020
Strumigenys denticulata	65	0.449	0.021	0.659	0.040	0.516	0.034	0.731	0.056	0.571	0.054	0.400	0.018	0.089	0.004	0.221	0.012
Strumigenys sanctipauli	6	0.987	0.034	0.612	0.039	0.423	0.031	0.905	0.041	0.835	0.067	0.314	0.025	0.101	0.004	0.221	0.031
Strumigenys schmalzi	2	0.409	0.041	0.834	0.086	0.647	0.015	0.602	0.080	0.630	0.073	0.608	0.041	0.049	0.005	0.245	0.025
Wasmannia affinis	87	0.565	0.041	0.717	0.123	0.755	0.067	0.841	0.090	0.728	0.064	0.165	0.032	0.187	0.018	0.250	0.026

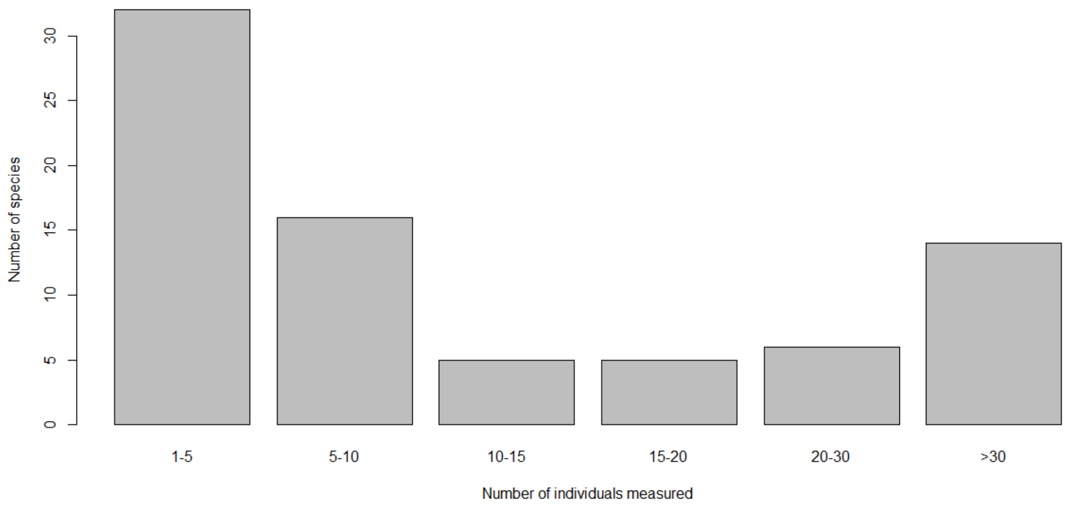

Supplementary Figure S2. Histogram of number of individuals with traits (eight) measured per species

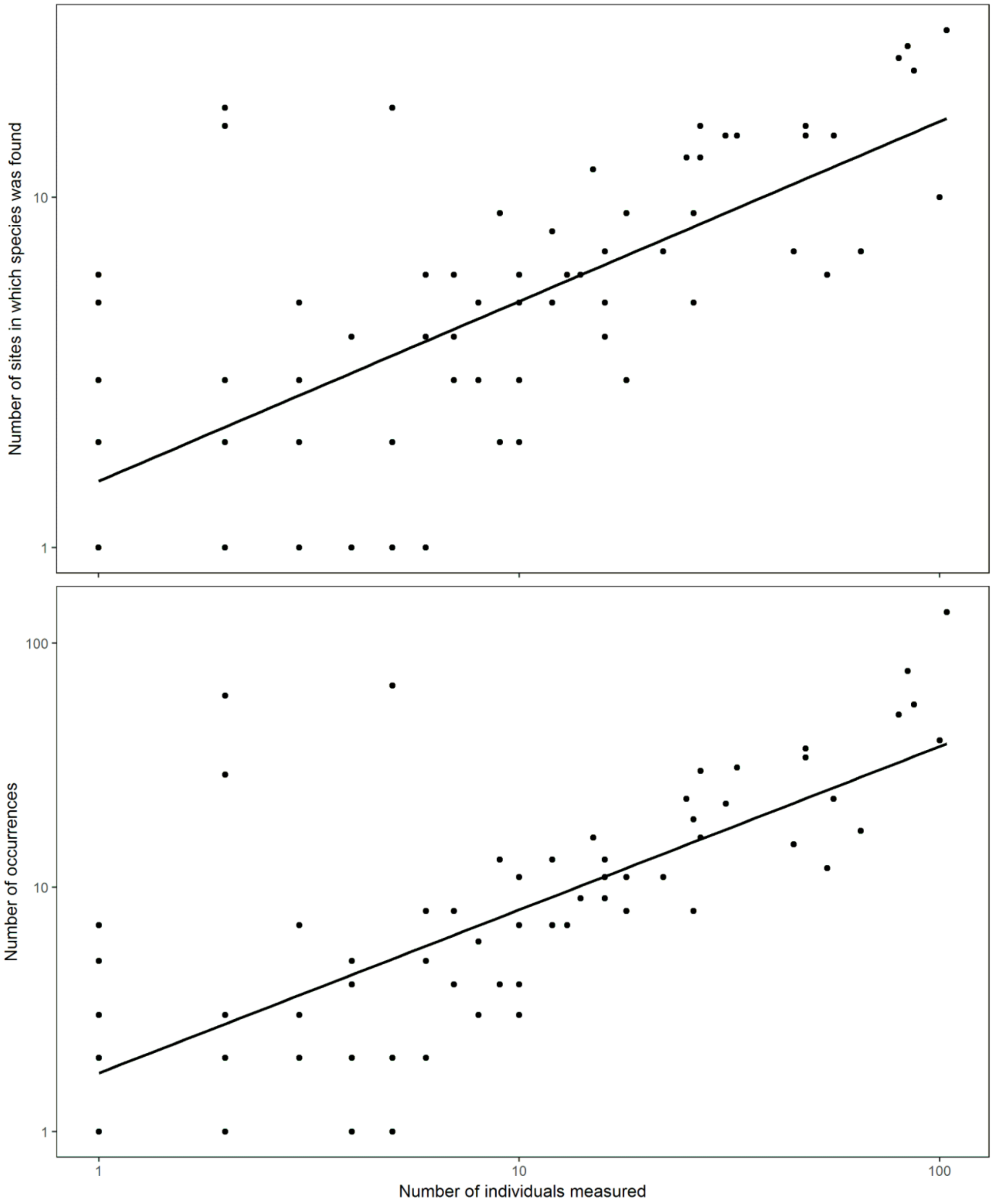

Supplementary Figure S3. Correlation between number of individuals measured, number of occurrences and plots in which species occurred. Black line represents linear regression between variables, scales were log transformed.

Supplementary Table S4. Moran's I values and the significance values (p) of Monte Carlo permutation for spatial autocorrelation between the coordinates' dissimilarity matrix and each response variable's dissimilarity matrix of ant communities.

Variable	***Moran's I***	***p***
Richness	-0.020	0.502
FRic	0.104	0.194
FRed	0.007	0.448
Rao Q's	-0.050	0.636

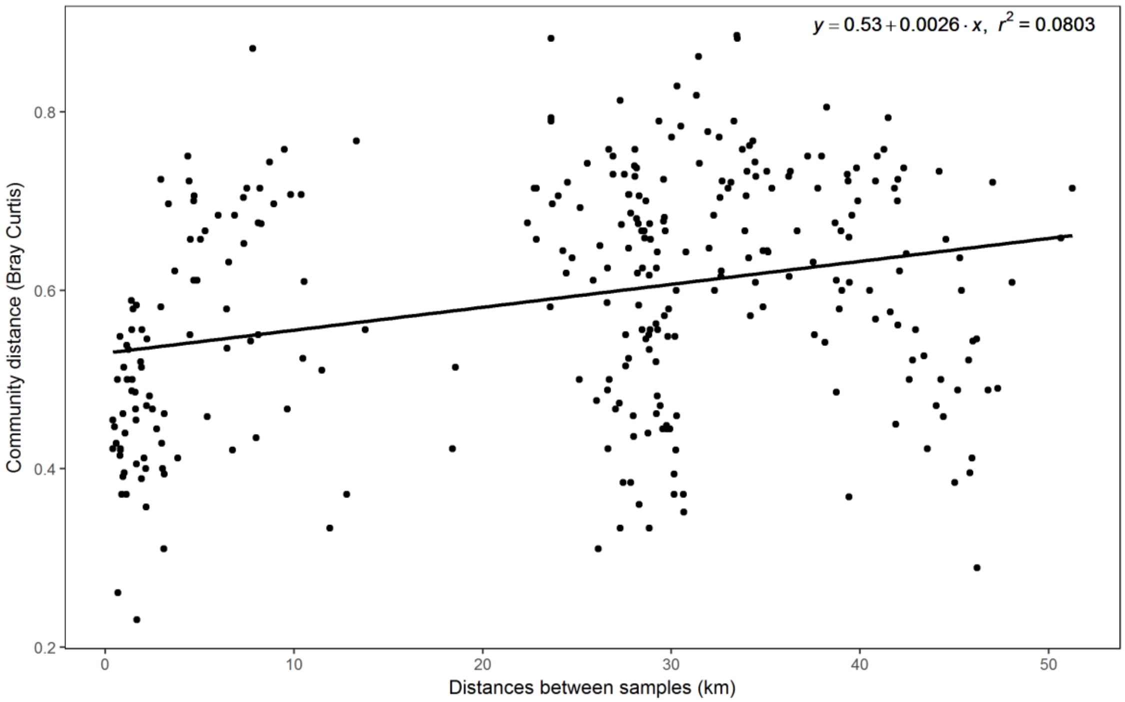

Supplementary Figure S5. . Spatial auto correlation between communities (Bray-Curtis distance) and distances between samples. Black line represents linear regression between variables.
